# Ultrasound-Assisted Extrusion Compounding of Nano Clay/Polypropylene Nano Compounds

**DOI:** 10.3390/polym16172426

**Published:** 2024-08-27

**Authors:** Gaston Francucci, Elena Rodriguez, María Eugenia Rodriguez

**Affiliations:** Eurecat—Centre Tecnològic de Catalunya, Unit of Polymeric and Composites Processes, Av. Universitat Autònoma, 23, 08290 Cerdanyola del Vallès, Spain; gastonfrancucci@gmail.com (G.F.); elena.rodriguez@eurecat.org (E.R.)

**Keywords:** compounding, ultrasound, twin screw extruder, thermoplastic, dispersion, additives, nano clays, barrier properties

## Abstract

The incorporation of nanoparticles can significantly enhance the properties of polymers. However, the industrial production of nanocomposites presents a technological challenge in achieving the proper dispersion of nanoparticles within the polymer matrix. In this work, a novel device is presented that can be seamlessly integrated with standard twin-screw extruders, enabling the application of ultrasonic vibration to molten polymeric material. The primary objective of this study is to experimentally validate the effectiveness of this technology in improving the dispersion of nanoparticles. To accomplish this, a comparative analysis was carried out between nanocomposites obtained through conventional compounding extrusion and those processed with the assistance of ultrasonic vibrations. The nanocomposites under investigation consist of a polypropylene (PP) matrix reinforced with nano clays (Cloisite 20A) at a target loading ratio of 5% by weight. To comprehensively evaluate the impact of the ultrasound-assisted compounding, various key properties were assessed, such as the melt flow index (MFI) to characterize the flow behavior, mechanical properties to evaluate the structural performance, oxygen barrier properties to assess potential gas permeability, and microstructure analysis using Scanning Electron Microscopy (SEM) for detailed morphology characterization. The results suggested an improvement in nanoparticle dispersion when using the ultrasound device, particularly when the intensity was adjusted to 60%.

## 1. Introduction

The excellent performance of polymer nanocomposites is attributed to the high surface-to-volume ratio of nanoparticles, which leads to a significant increase in the polymer/particle interfacial area. Thus, very low levels of nanoparticle loading can significantly modify the intrinsic properties of a polymer [1]. However, effectively reinforcing polymers with nanoparticles on an industrial scale remains a technological challenge that has not been fully overcome to date [2]. The potential of nanoparticles has been limited by difficulties associated with their dispersion in the polymer matrix, which directly affects the compound’s properties. This is because the dimension of the reinforcement must be on a molecular or atomic scale to effectively strengthen the matrix [3]. Nanoparticles tend to form agglomerates due to their typically low compatibility with the polymer matrix and their extremely large specific surface area. These factors, combined with the macromolecular nature of polymers, make nanoparticle dispersion a complex technological challenge. Typically, nanoparticle functionalization [4] and appropriate dispersion techniques are required, among which extrusion compounding is the only suitable method for large-scale production and, therefore, of great industrial interest [5]. Twin-screw extruders are often effective in achieving the good dispersion of micrometer-sized particles, but the mixing mechanisms in these machines are usually insufficient to ensure a high degree of nanoparticle dispersion. Ultrasound irradiation in molten polymers has the potential to address this problem by allowing the temperature of the molten polymer to increase and its viscosity to change [6], breaking up particle aggregates through high-energy ultrasonic waves [7], increasing the free volume and reducing molecular entanglements [8], and generating polymer radicals that can combine with nanoparticles and/or the modifying agent, acting as an intrinsic compatibilizing agent [9]. On the other hand, severe ultrasound irradiation can lead to polymer degradation, causing a significant reduction in intrinsic viscosity or molecular weight [10].

Similarly, ultrasonic extrusion technology has been applied in many fields including building ceramics, where it has brought several advantages, such as decreasing the friction between the clay and the die, decreasing the pressure in the die (and increasing the extrusion output), and positively influencing the water absorption and strength of ceramics (and reducing scatter in their properties) [11].

This work presents the ultrasound-assisted extrusion compounding process using a device patented by EURECAT [12], designed to be coupled with conventional twin-screw extruders. As an example of its use, the production and characterization of nano compounds based on PP and nano clays using traditional extrusion compounding and ultrasound-assisted processes are shown. Nano clays are widely used as nano reinforcements of polymeric materials to improve the mechanical properties and to increase the barrier properties of polymers. Their main advantage over other nano reinforcements is their low cost [13,14,15,16].

It is worth noting the unique morphology of clays and how it can be modified to affect the properties of the nano compound in different ways. The crystalline structure of nano clays consists of two-dimensional layers where a central octahedral layer of alumina or magnesia is bound to two external tetrahedrons of silica in such a way that the oxygen ions of the octahedral layer also belong to the tetrahedral layers [17]. The aluminosilicate layers are stacked on top of each other, with cations (Na^+^, K^+^, Mg^+^, Ca^+^) present between them. These cations are typically exchanged through an ion exchange reaction with positively charged surfactants, such as alkylammonium or phosphonium cations with long alkyl chains [18,19]. Consequently, the basal spacing of the layers increases, reducing the surface energy of the particles and increasing the compatibility with polymers. When these modified clays are mixed with polymers, three different morphologies or degrees of dispersion can be achieved: immiscible, intercalated, and miscible or exfoliated [20]. The first case occurs when the mixing process is insufficient, resulting in the clay forming agglomerates of micrometer-sized particles. The intercalated state implies that while the clays retain their morphology of stacked parallel layers, some polymer molecules manage to enter the galleries between them, improving the polymer-reinforcement interaction and, therefore, the material’s properties. If the mixing is highly effective, the optimal degree of dispersion can be achieved, which is the complete exfoliation of the clay, where individual sheets are fully separated and dispersed within the polymer matrix.

US treatment during the extrusion compounding process has been shown to be effective in improving the dispersion of the nano clays and overall nanocomposites’ properties, as summarized in Table 1.

Regarding the effect of ultrasonic treatment on polymer morphology, it was reported that the breakdown of agglomerates due to US treatment led to a faster nucleation rate and a smaller size of HDPE spherulites, suggesting that the presence of many smaller clay particles acted as nucleation sites. HDPE/clay composites at a loading of 5 wt% were reported to have lower crystallinity than untreated composites, which was ascribed to a better compatibility between HDPE and clay, leading to a restriction of the molecular mobility of the polymer [24].

## 2. Materials and Methods

### 2.1. Materials

The polymer used was a PP from Borealis (Vienna, Austria), code BF970MO, which is a heterophasic copolymer characterized by an optimum combination of a very high stiffness and high impact strength. The nano filler used to produce compounds was a modified clay supplied as a powder, Cloisite 20A, which is a phyllosilicate recommended as a flame-retardant synergist in halogen-free flame-retardant thermoplastics and to improve the physical and barrier properties in thermoplastic compounds. Table 2 presents the properties of both the polymer and filler, as reported in the technical data sheets. The targeted final composition of the compounds was 95% PP/5% Cloisite 20A (by weight).

### 2.2. Ultrasound-Assisted Extrusion Compounding

#### 2.2.1. Compounding

The compounding process was carried out in a laboratory extruder ZSK 18 MEGAlab from Coperion (Stuttgart, Germany). This machine has co-rotating intermeshed screws, and the specifications are summarized in Table 3.

The extrusion parameters that were used are detailed in Table 4, which were selected based on recommendations extracted from the literature and also a preliminary degradation study carried out on the compounds.

Recommendations in the literature:

- Feeding protocol. Dissolution from a masterbatch yields better results than feeding the clays through the side feeder (more residence time in the mixing zones) [32].
- Feeding rate. Low feeding rates (≈3 kg/h) increase the residence time of the material in the mixing zones, improving clay dispersion [33,34,35]. However, very low feeding rates (≈1 kg/h) can lead to excessively long residence times and the degradation of the organic modifiers of the clays, reducing the mixing effectiveness [36,37,38].
- Heating ramp. This variable was found not to significantly affect the degree of dispersion [39], but some works suggest working at the lower side of the processing temperature range because a higher melt viscosity would increase the shear forces on the material, which can help break down clay agglomerates [40,41].
- Screw rotation speed. Within the typical range of variation (typically 50–500 min^−1^), an increase in speed improves the dispersion state of the clay due to increased shear [32]. However, it has been reported that in the domain of very high speeds (1000–4000 min^−1^), the degree of exfoliation may decrease due to high-speed matrix degradation, resulting in a drop in viscosity and less efficient exfoliation [42,43].

Degradation study of the polymer matrix

During the extrusion process, the polymeric material is subjected to high temperatures and shear stresses, resulting in the thermo-mechanical degradation of the polymer matrix and also of the organic cations used to chemically modify the nano clay. The processing parameters that significantly affect the degradation are as follows:

- Temperature (T): Higher temperatures lead to increased degradation.
- Feed rate (Q): Lower feed rates result in a longer residence time (t_R_) and, consequently, greater degradation.
- Screw rotation speed (N): Higher rotation speeds impose greater shear stresses on the polymer, leading to increased heat generation and degradation.

In fact, the degradation (DEG) is proportional to the flow stress imparted on the molten material (σ_Y_), which is proportional to the specific mechanical energy (SME) experienced during the extrusion process. The SME is inversely proportional to the Q/N ratio:

(1) $$DEG\;\propto \sigma_{Y}\;\propto SME=\frac{P_{motor}\tau_{R}N_{R}}{Q}$$

In Equation (1) [44], SME (kWh/kg) represents the specific mechanical energy, P_motor_ denotes the motor power (W), and τ_R_ is the relative torque recorded in the process (%), which is the process torque divided by the maximum torque of the machine. N_R_ represents the relative rotation speed (the screw rotation speed divided by the maximum machine rotation speed), and Q is the feeding mass flow rate (kg/h).

It is important to carry out the compounding extrusion process by adjusting the process parameters in such a way as to maintain the degree of polymer degradation below predetermined acceptable limits. The procedure for determining the operating limit based on the degradation condition involved conducting extrusion tests at different temperatures (180 °C and 220 °C) using pairs of Q and N values selected to evaluate three operating conditions:

- Minimum specific mechanical energy (SME): Q/N = 2.5
- Intermediate specific mechanical energy (SME): Q/N = 1.75
- Maximum specific mechanical energy (SME): Q/N = 1

The polymeric material used for these tests was formulated to be identical to the polymer matrix present in the compounds under study, consisting of

- 74.9% virgin PP.
- 25.1% masterbatch containing 80% PP/20% Cloisite 20A.

Melt Flow Index tests were conducted on the materials obtained under each process condition according to the UNE EN ISO 1133-1 standard [45], using the parameters reported in the PP technical data sheet (T = 230 °C, weight = 2.16 kg).

#### 2.2.2. Ultrasound Device

**Figure 1:**
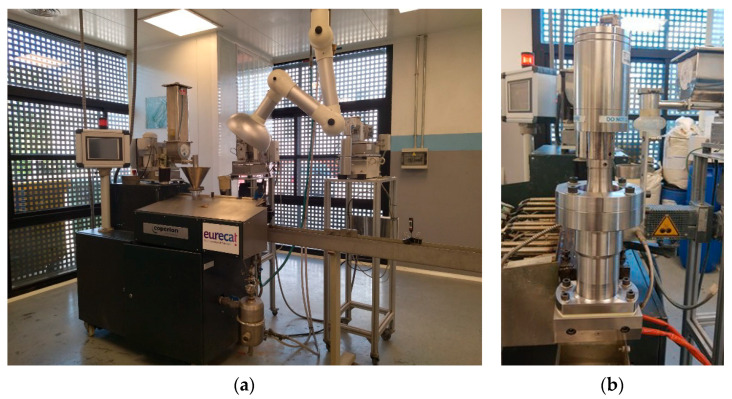
Twin screw extruder (**a**) and US device (**b**) attached to it.

The ultrasound system has a power of 1500 W, operates at a fixed frequency of 20 kHz, and allows for reaching maximum amplitudes of 74.3 μm. It is controlled by ten levels of intensity (each 10% increase in intensity corresponds to a 7.43 μm increase in oscillation amplitude). The operation frequency was determined after thorough R&D during the US device design and manufacture, 20 kHz being the one that ensured the most stable continuous operation. The device is coupled to the extruder through a specifically designed die that is screwed onto the end of the extruder. It contains four air cooling channels to prevent the temperature of the sonotrode from increasing and damaging the piezoelectric material. The sonotrode can be placed in three different positions, penetrating into the channel through which the material flows at different fixed depths of 4 and 6 mm. Figure 1 shows photographs of the manufacturing equipment.

Subjecting the molten polymer to ultrasonic vibrations often leads to its degradation through the formation of macro radicals. It is important to keep the level of degradation below acceptable limits, allowing for a favorable balance between the benefits of ultrasound (improved dispersion of nanoparticles) and its drawbacks (polymer degradation). For this reason, tests were conducted to assess the degree of degradation generated by the ultrasonic treatment on the polymer matrix of the compounds. The material was extruded at the condition established in the previous section, and the ultrasound intensity was varied from 0% to 100% using the US die in both configurations (4 and 6 mm). MFI tests were conducted on the resulting material to assess the degradation in the materials (following the UNE EN ISO 1133-1 standard, with the same conditions as those used in the previous section).

### 2.3. Compounds’ Characterization

The obtained materials were thoroughly characterized to evaluate the effect of the US device on the nano clay’s dispersion and the compound’s properties. The following tests were conducted:

- Thermo-gravimetric analysis, TGA (in compliance with the UNE EN ISO 11358-1 standard [46]), was used to determine the composition of the compounds and their thermal stability (degradation temperatures). TGA was carried out in a TA Instruments (New Castle, DE, USA) thermogravimetric analyzer. Approximately 10 mg of each mixture was placed in a nitrogen atmosphere and heated at 10 °C/min over a temperature range of 25–1000 °C. The nitrogen flow over the measurement cell was 50 mL/min.
- The MFI was determined in a plastometer (model 7023.000, CEAST (https://www.instron.com/en/products/materials-testing-software/ceast-software, accessed on 23 August 2024)) in accordance with the UNE EN ISO 1133-1 standard to evaluate the fluidity of the compounds. The test conditions were set at a load of 2.160 kg and a temperature of 230 °C for all the materials under study.
- Flexural tests were carried out to characterize the mechanical properties using an INSTRON (Norwood, MA, USA) Universal Testing machine, according to the ISO 178 standard [47]. The specimens (un-notched) exhibited dimensions of 80 × 10 × 4 mm^3^ and were produced directly by injection molding using the extruded pelletized material. The cross-head displacement speed was 2 mm/min.
- Oxygen permeability tests (according to ASTM D3985-17 standard [48]) under conditions of 23 °C/0% relative humidity (RH) were conducted to characterize the barrier properties of films manufactured with the different materials under study.
- Scanning Electron Microscopy (SEM) images were obtained using a (FE-SEM) ZEISS (Jena, Germany) Ultraplus Microscope. The samples were coated with a thin layer of gold, the tests were conducted under high vacuum, the acceleration voltage was varied between 5 and 15 A, and the electron beam current was adjusted to minimize charging.

## 3. Results

### 3.1. Degradation Assessment during the Extrusion Compounding Process

Table 5 presents the results obtained when using a process temperature of 220 °C, where the term “DEG” denotes “degradation” in cases where excessive fluidity prevented the correct measurement of MFI. Please note that the units of N (rpm) were not changed to rph to match the temporal units of Q (g/h) in order to obtain values of R that are visually easy to compare. Considering that the MFI of virgin PP was found to be 9.6 g/10 min and the MFI of the masterbatch was 14.2 g/10 min, the MFI of the mixture was estimated to be 10.75 g/10 min (by the rule of mixtures). Therefore, the MFI values obtained from the tests at 220 °C are considered excessively high, indicating unacceptable polymer degradation.

**Figure 2:**
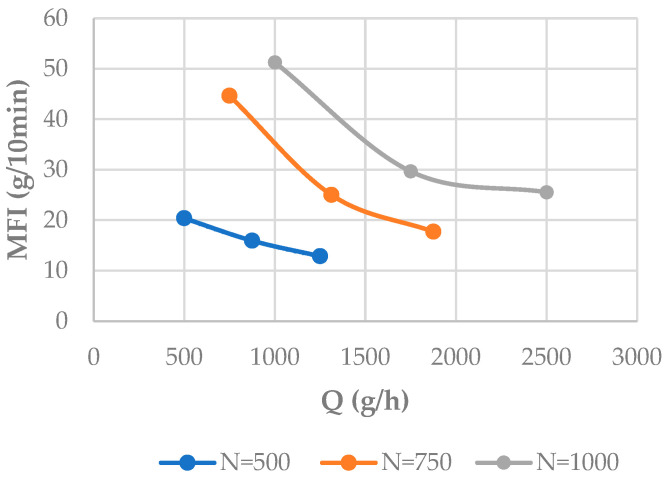
Effect of the feeding rate and screw rotation speed on the MFI.

The results obtained at 180 °C are presented in Table 6 and schematically represented in Figure 2. As expected, the material degradation increases with higher N values and lower Q values.

**Figure 3:**
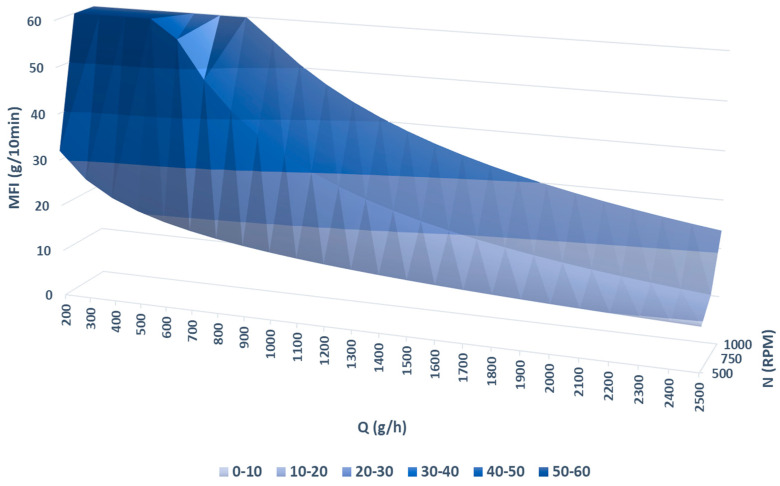
MFI as a function of N and Q.

The dependence of MFI on the feed rate for each screw rotation speed was fitted with a power-law-type model using data analysis and graphing software, and the parameters are detailed in Table 7. This model was used to interpolate the values and generate a surface plot that illustrates the evolution of MFI when modifying both the feed rate and screw rotation speed (Figure 3).

**Figure 4:**
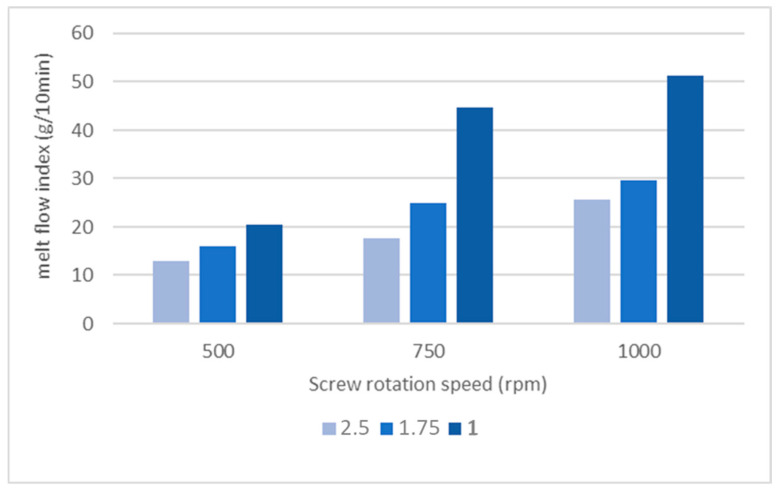
MFI as a function of Q/N.

While the Q/N ratio is inversely proportional to the specific mechanical energy (SME) experienced by the polymer during the extrusion process, it has been observed that the MFI value of the material does not remain constant when the Q/N ratio is held fixed. Even with a constant Q/N ratio, the material degradation is higher when both N and Q increase proportionally, as shown in Figure 4. These results suggest that the effect of N is more significant than that of Q in the degradation of the material.

To determine the limit imposed by material degradation within the operating window, a maximum value of MFI representing the allowable maximum degradation generated in the compounding extrusion process was established. Then, using the power-law expressions from Table 7, the minimum allowable feed rate (Q_limit_) can be calculated for each screw rotation speed (Table 8).

**Figure 5:**
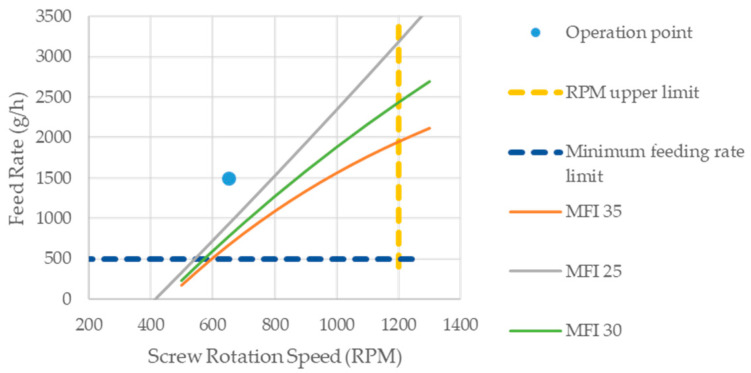
Processing window defined by the machine operative limits and different degradation thresholds.

It should be noted that the relative torque τ_R_ recorded in the extrusion tests remained nearly unchanged (with a variation of less than 4%) for all the tests. Therefore, the specific mechanical energy is considered directly dependent on the Q/N ratio used in the compounding process. The pairs of values (N. Q_limit)_ were plotted in the operating window and fitted with an exponential function (Table 9) representing the operational limit established by material degradation. Figure 5 displays the processing windows at 180 °C for each selected maximum MFI value. As expected, the degradation curves do not exhibit a linear trend because the MFI varies when modifying Q and N, even if the Q/N ratio remains constant. A maximum MFI value of 25 g/10 min was chosen as the admissible maximum degradation limit, so the operation point (shown in Figure 4) was set in the “safe zone” defined by the MFI 25 curve.

### 3.2. Degradation Assessment during the Ultrasound-Assisted Extrusion Compounding Process

**Figure 6:**
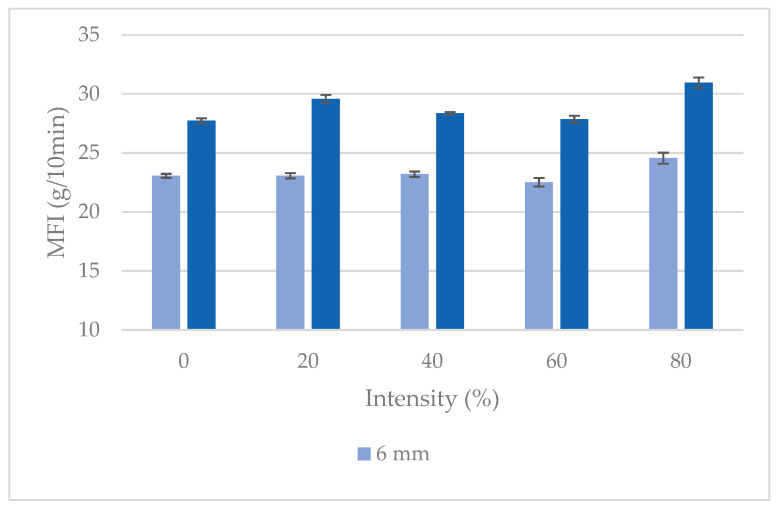
MFI of compounds processed under different US conditions.

The material was extruded at the operation point defined in the previous section and subjected to different US conditions at the die. The MFIs of the resulting material are presented in Figure 6. It can be observed that the MFI of the material processed using the sonotrode at the configuration of 4 mm in depth was always higher than the MFI obtained using the sonotrode at 6 mm. The MFI did not significantly vary for amplitudes between 0% and 60%, and the first significant increase was observed when using 80% intensity. The results for 100% intensity are not included in Figure 6 due to excessive and visually detectable degradation during the process. After this analysis, it was decided to carry on the processing of compounds using US intensities up to 60%.

### 3.3. Compound Characterization

**Figure 7:**
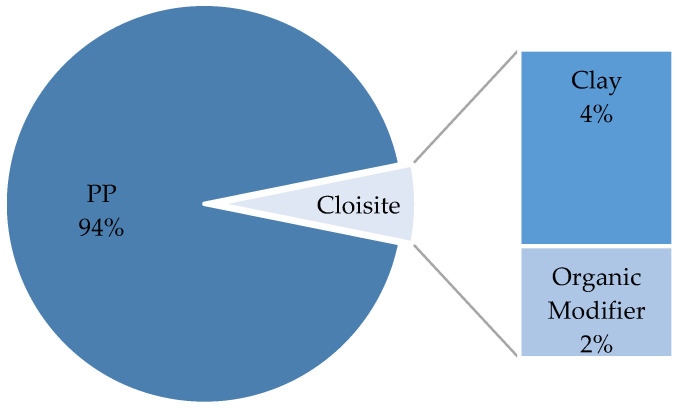
Mass percent composition of the compounds.

The final composition of the compound was verified through TGA tests and resulted in 96% PP and 6% Cloisite 20A. Additionally, the commercial product Cloisite 20A is 64% clay and 36% organic modifier (these data are provided in the product datasheet, but it was also verified by the TGA of pure Cloisite 20A), so the compounds contained a final clay content of 4%. Figure 7 presents the final composition of the compounds under study.

The addition of nano clays to thermoplastic polymers has been found to have a significant effect on the viscosity of the polymer. Studies have shown that the clay spacing in the composites affects the viscosity. When there is a strong interaction between the polymer and the nano clay, the addition of the clay can greatly increase the viscosity of the polymer [49]. This increase in viscosity can be attributed to the formation of a nano clay network and the exchange reaction between the polymer chains [50]. Therefore, a reduction in the MFI should indicate an improvement in the dispersion degree of the nanoparticles (more surface area interacting with the polymer molecules, reducing the material’s fluidity), while an increase in MFI could be associated with poor nanoparticles dispersion and excessive degradation of the polymer matrix (reducing its molecular weight and, therefore, its melt viscosity).

**Figure 8:**
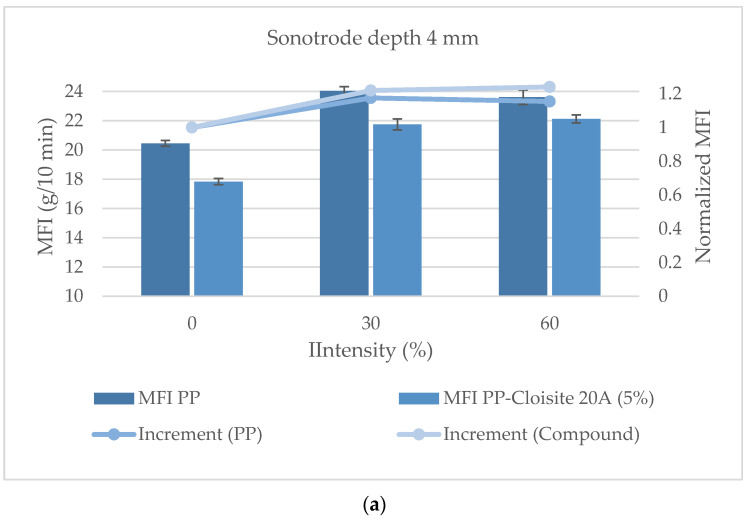

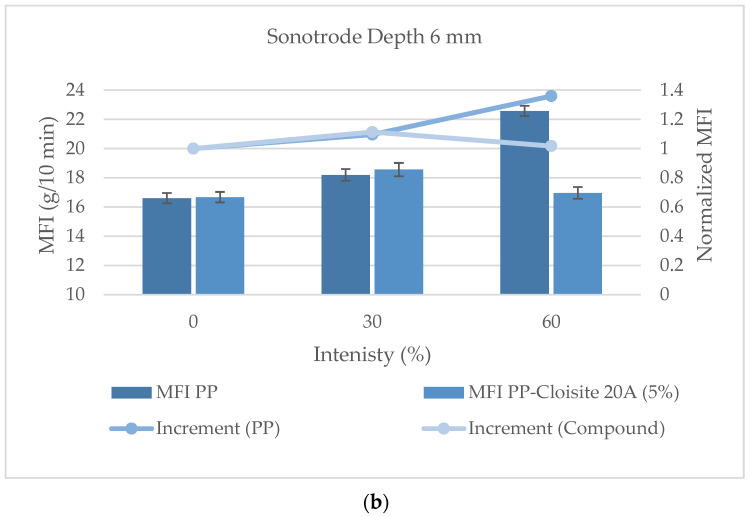
MFI of compounds processed with the sonotrode at 4 mm (**a**) and 6 mm (**b**).

Figure 8 shows the results from the MFI tests. When the sonotrode tip depth was 4 mm, the fluidity of the material increased as the US intensity increased. This might be due to the excessive degradation of the polymer. No real differences could be observed between 30% and 60% of intensity, and the responses to the US treatment of the polymer and the nano compounds were the same. Again, excessive degradation could be hindering any beneficial effect of the US device on the dispersion degree of the nano particles. When the sonotrode was used at a depth of 6 mm, the results were different. While a first increment in MFI was observed for both the PP matrix and the nano compound treated with 30% US intensity, a further increase in intensity produced a significant decrease in the MFI of the nano compounds, but the MFI of the PP kept rising. The hypothesis in this case is that, although polymer degradation increases from 30% to 60% of US intensity (as demonstrated by the increase in the MFI of PP), the US treatment carried out under these conditions (6 mm in depth of the sonotrode and 60% US intensity) was effective in improving the dispersion of the nano clays.

After analyzing the MFI results, the samples processed with the sonotrode tip depth set at 4 mm were not further evaluated, and the following characterization has been carried out on compounds obtained with the 6 mm depth configuration.

The use of nano clays as fillers in polymer nanocomposites has been shown to enhance the thermal barrier of materials [51,52,53]. However, it is understood that this improvement depends on the degree of dispersion of nano clays in the polymer matrix. Studies have shown that nano clay-based nanocomposites demonstrate a reduced rate of heat release and higher thermal stability compared to the virgin polymer or systems where the clay is not dispersed at the nano level [54]. For example, the thermal stability improvement in polymer/montmorillonite (MMT) nanocomposites has been attributed to the “labyrinth effect” and the dispersity of MMT [55].

**Figure 9:**
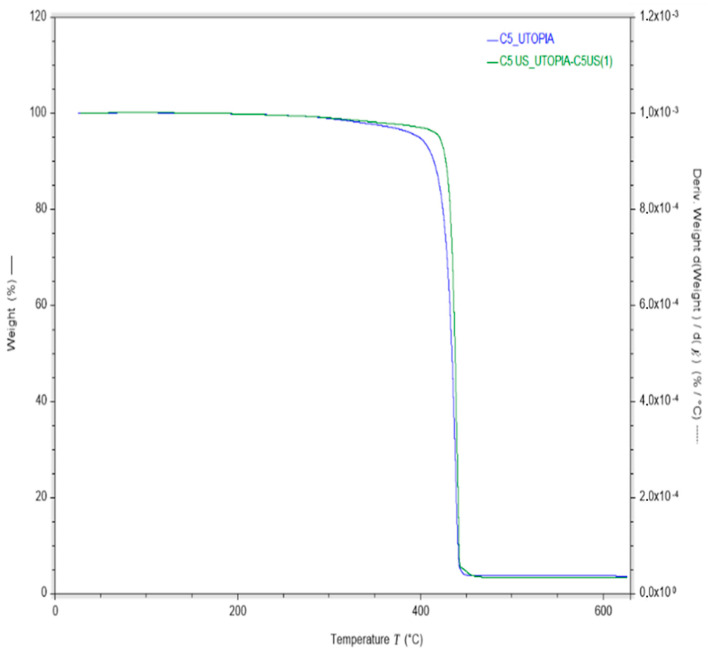
TGA curves of the nano compounds under study.

Figure 9 shows the TGA curves for the compounds obtained with (C5 US) and without (C5) ultrasonic treatment during the extrusion compounding process. The compounds C5 US characterized by TGA were the ones treated with 60% US intensity after the good results found in the MFI tests. Evidently, the ultrasonic-treated compound has better thermal stability than the one processed by traditional extrusion compounding. The onset of the mass loss of C5 is 330 °C, while in C5 US, it is shifted to higher temperatures, over 400 °C, and the degradation temperature of C5 is 400 °C, whereas in C5 US, it is 425 °C. This improvement given by the ultrasonic treatment suggests that the nano clays are better dispersed in the polymer matrix in comparison to the non-sonicated compounds.

**Figure 10:**
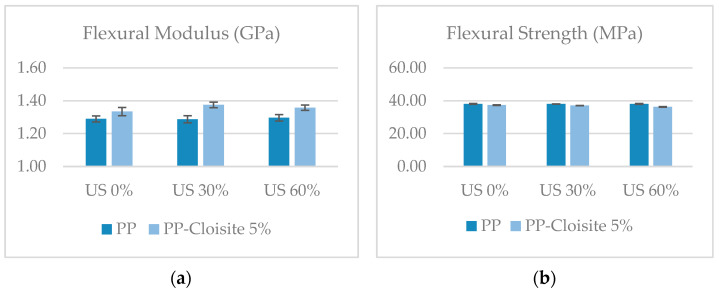
Flexural properties of the compounds. (**a**) Flexural modulus. (**b**) Flexural strength.

Mechanical properties were characterized by means of flexural tests. Figure 10 shows the flexural strength and stiffness of nano compounds produced with different US conditions. The addition of nanoparticles slightly increased the rigidity (flexural modulus) of the PP (about 3%). When the US is on, the improvement in the modulus is marginally higher than it is when the US is off (6% with the US on). No significant differences could be observed in the flexural modulus among samples processed using 30% and 60% intensity. Regarding the flexural strength, the addition of nano particles slightly decreased this property, and no significant differences could be found in the samples processed with and without US treatment, varying the US intensity. These findings are consistent with other results reported in the literature. Several studies have reported a decrease in the strength of thermoplastic polymers with the addition of nano clays. For example, in a study by Assaedi et al. [52], the compressive strength of geopolymer paste initially improved with the addition of 2.0 wt% nano clay, but it decreased when the clay content was increased to 3.0 wt%. Similarly, in the investigation conducted by Fereydoon et al. [50], the flexural strength of thermoplastic resin decreased with the incorporation of 7 wt% silica and zirconia nanoparticles. Additionally, the tensile strength and flexural strength of epoxy composites reinforced with curauá fibers and organophilic clay were found to decrease with the addition of 5 wt% nano clay [56]. On the other hand, the addition of nano clays to thermoplastic polymers has been found to increase the stiffness of the resulting composites in several studies. Khan et al. [57] demonstrated that the addition of solvent-exfoliated graphene to polyurethane resulted in composites with increased stiffness. Similarly, Amjad et al. [58] reported that the incorporation of nano clay in thermoset- and thermoplastic-based natural fiber composites led to an increase in stiffness. Some other studies have also reported an increase in stiffness with the addition of nano clays to thermoplastic polymers, including cellulose nanocrystal-reinforced starch films [59], recycled poly(ethylene terephthalate) composites [60], epoxy/thermoplastic/organoclay systems [61], and polyamide 12 composites [62].

**Figure 11:**
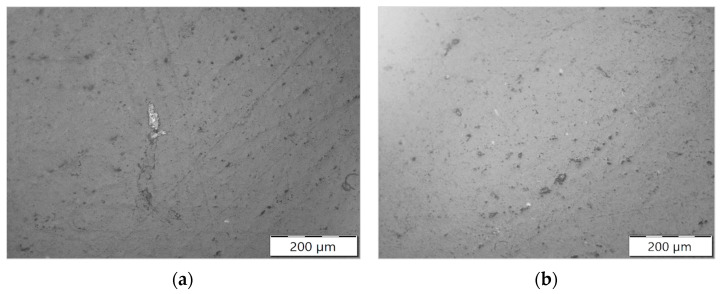
SEM images of the nano compounds. (**a**) Traditional extrusion compounding. (**b**) Ultrasound-assisted extrusion compounding.

Micrographs of the compound’s microstructure were obtained by scanning electron microscopy (SEM). SEM provides high-resolution images of the surface morphology of materials, allowing for the observation of the dispersion state of fillers in the polymer matrix [63]. It can provide information about the size, shape, and distribution of clay particles within the nanocomposite. SEM can also be used to analyze the interface between the clay particles and the polymer matrix. providing insights into the adhesion and interaction between the two components [64]. However, SEM has a lower resolution compared to TEM (Transmission Electron Microscopy), since TEM provides higher magnification and allows for the direct visualization of the microstructure, including the arrangement and dispersion of clay particles at the nanoscale [65]. However, TEM requires time-consuming sample preparation, and the high-energy electron beam used in TEM can potentially damage the polymer matrix [66]. More than 22 SEM images were obtained of the nanocompounds obtained with and without the ultrasonic treatment. Figure 11 shows the two images that contained the larger agglomerates among the batch. It can be seen that the size of the clay aggregates is much larger in the non-sonicated sample (Figure 11a) than in the one subjected to the ultrasonic treatment (Figure 11b).

**Figure 12:**
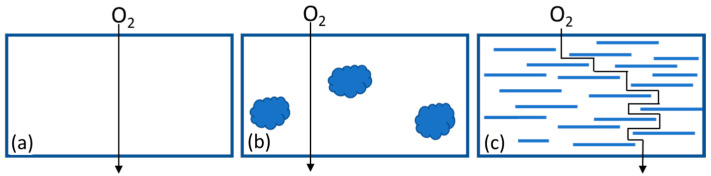
Possible morphologies. (**a**) Polymer matrix. (**b**) Nanocomposite with clay agglomerates. (**c**) Nanocomposites with intercalated or exfoliated clay.

The oxygen barrier properties test is suitable for indirectly assessing the degree of dispersion of nano clays in PP, since this property is less affected by polymer degradation. Also, many industrial applications of nanocomposites, particularly in the packaging industry, rely mainly on the materials’ barrier properties and do not require a significant increase in the mechanical properties of the base materials. Theoretically, if the dispersion of the nano clay layers is effective, it is expected that the material will have lower oxygen permeability due to the increased tortuosity of the diffusive path for gas molecules through the sample thickness, as schematically shown in Figure 12.

Three materials were chosen for the O_2_ permeability tests: neat PP (baseline) and PP additivated with nano clays (5%) without US treatment and with US treatment at an intensity of 60% (being the one material that showed better performance in previous tests). The oxygen barrier properties were measured in 70 μm thick films extruded with pellets of the mentioned materials. The results are presented in Table 10. The addition of clays without the use of the ultrasound system worsened the barrier properties of PP, which can be explained by imperfections generated by clay agglomerates on the material surface that could facilitate the passage of oxygen molecules through the sample (as shown in Figure 12a). However, when using the ultrasound device, the permeability coefficient of the material was reduced even below the value of virgin PP, indicating that the device was effective in breaking up agglomerates and improving the dispersion of nano clays in the polymer matrix.

## 4. Conclusions

This work analyzed the performance of an ultrasound device that is attached after the twin screw extruder for improving nano clay dispersion in a thermoplastic polymer. The compounds consisted of 94% PP and 6% Cloisite 20A. The improvement in clay dispersion was confirmed by the results obtained in many characterization tests. The use of scanning electron microscopy (SEM) allowed for the observation of the surface morphology and distribution of clay aggregates, highlighting the larger agglomerates in the non-sonicated sample compared to the ultrasound-treated sample. The melt flow index (MFI) tests indicated that a reduction in MFI was associated with an improved dispersion of the nanoparticles. Thermal stability tests revealed that the ultrasonic treatment led to better thermal stability, indicating improved dispersion of the clay nanoparticles. Flexural tests demonstrated a slight increase in stiffness and a slight decrease in flexural strength with the addition of nanoparticles, consistent with previous literature findings. Oxygen permeability tests indicated that the dispersion of clay layers effectively reduced the oxygen permeability of the nanocomposites, enhancing their barrier properties.

Regarding the operative parameters, the best results were obtained by placing the sonotrode at a depth of 6 mm in the molten flow chamber and using an ultrasound intensity of 60% (a US amplitude of 44.58 μm). The configuration of 4 mm in depth of the sonotrode was found to be harsh for the material, causing excessive degradation, as well as using intensities over 60% (for both sonotrode locations).

## 5. Patents

The research project’s main goal has been to validate the patented technology (Ultrasonic device for a polymer extrusion machine (EP EP3603933A1)) from its current state, TRL 4 to TRL 7, allowing for a major step increase in the technological maturity of the technology.

## Figures and Tables

**Table 1:** Effect of ultrasonic treatment on clay/polymer composites.

Polymer	Clay Morphology	Improvement in Properties	Ref.
PS/LDPE	Exfoliated	Young’s modulus, elongation at break, yield stress	[21,22,23]
HDPE	Intercalated/partially exfoliated	Increase in Young’s modulus, tensile strength, yield stress, elongation at break, and Izod impact strength	[24]
PA6	Intercalated/partially exfoliated	Young’s modulus, elongation at break, yield stress, impact strength	[25,26]
PMMA	Intercalated/partially exfoliated	Storage modulus, thermal stability	[27]
EVA	Intercalated	Young’s modulus	[28]
PP	Intercalated	Elongation at break and toughness	[29,30]
PP/PP-g-MA	Immiscible/No effect observed	Increase in Young’s modulus, tensile strength, elongation at break, and Izod impact strength	[31]
HDPE/HDPEg-MA	Immiscible/No effect observed	Young’s modulus	[32]

**Table 2:** Materials’ properties.

Property	Value	Units
Polypropylene (PP)		
Density	905	kg/m^3^
Melt flow rate (230 °C/2.16 kg)	20	g/10 min
Flexural modulus	1450	MPa
Tensile modulus (1 mm/min)	1500	MPa
Tensile strain at yield (50 mm/min)	5	%
Tensile stress at yield (50 mm/min)	27	MPa
Cloisite 20A		
Bulk density	350	kg/m^3^
Density (20 °C)	1.80	g/cm^3^
Particle size	<10	μm
Moisture content	<2.5	%
Lamellar spacing (XRD, d001)	2.7	nm

**Table 3:** Extruder main specs.

Heated zones	8	Maximum feed rate (Q)	40 Kg/h
Screwouter diameter (D)	18 mm	Screw rotational speed (N)	0–1200
Screw length (L)	720 mm	L/D	40

**Table 4:** Extrusion compounding parameters.

Q	N	Temperature Profile							
g/h	rpm	°C							
1500	650	160	165	170	170	180	180	180	180

**Table 5:** MFI of compounds extruded at 220 °C.

T	N	Q	R = Q/N	MFI
°C	rpm	g/h		g/10 min
220	500	1250	2.5	55.26
		875	1.75	78.31
		500	1	DEG
	750	1875	2.5	69.22
		1312.5	1.75	DEG
		750	1	DEG
	1000	2500	2.5	97.87
		1750	1.75	DEG
		1000	1	DEG

**Table 6:** MFI of compounds extruded at 180 °C.

T	N	Q	R = Q/N	MFI
°C	rpm	g/h		g/10 min
180	500	1250	2.5	12.88
		875	1.75	15.94
		500	1	20.39
	750	1875	2.5	17.72
		1312.5	1.75	25.00
		750	1	44.66
	1000	2500	2.5	25.53
		1750	1.75	29.68
		1000	1	51.29

**Table 7:** Power law fitting parameters.

N	Fitting Expression
rpm	g/10 min
500	$MFI=420*Q^{-0.49}$
700	$MFI=38,020*Q^{-1.02}$
1000	$MFI=15,700*Q^{-0.83}$

**Table 8:** Minimum allowable feed rate for each screw rotation speed based on the established maximum MFI limit.

Max MFI	N (rpm)		
(g/10 min)	500	750	1000
35	132	851	1260
30	228	1107	1884
25	332	1324	2346
20	525	1648	3070

**Table 9:** Relationships between Q and N that lead to specific MFI values.

Max MFI	Q_limit_
(g/10 min)	(g/h)
35	$Q=-2140+5860*\left[1-e^{\left(-0.001*N\right)}\right]$
30	$Q=-1892+9681*\left[1-e^{\left(-0.0005*N\right)}\right]$
25	$Q=-1565-30,904*\left[1-{\;e}^{\left(0.00012*N\right)}\right]$
20	$Q=-1062-2630*\left[1-e^{\left(0.0009*N\right)}\right]$

**Table 10:** O_2_ barrier properties.

Sample ID	PP1600	NC1600	NC1660
Film photo	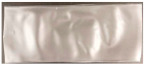	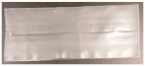	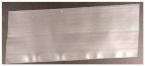
Compound	100% PP	95%PP/4%Cloisite	95%PP/4%Cloisite
US settings	US OFF	US OFF	US ON; intensity 60%.
O_2_ permeability coefficient (cm^3^ mm/m^2^ day atm)	113 ± 8.3	130 ± 6.6	109 ± 9.8

## Data Availability

Research data will be available upon request.
